# The Effect of Controlled Decompression for Severe Traumatic Brain Injury: A Randomized, Controlled Trial

**DOI:** 10.3389/fneur.2020.00107

**Published:** 2020-02-18

**Authors:** Junhui Chen, Mingchang Li, Lei Chen, Weiliang Chen, Chunlei Zhang, Yi Feng, Yuhai Wang, Qianxue Chen

**Affiliations:** ^1^Department of Neurosurgery, Renmin Hospital of Wuhan University, Wuhan, China; ^2^Department of Neurosurgery, Wuxi Clinical College of Anhui Medical University, 904th Hospital of Joint Logistic Support Force of PLA, Wuxi, China

**Keywords:** traumatic brain injury, controlled decompression, ICP, RCT, intraoperative brain swelling, delayed hematoma

## Abstract

**Background:** Experimental evidence has indicated the benefits of intraoperative controlled decompression for the treatment of severe traumatic brain injury (sTBI). Intraoperative rapid decompression (conventional decompression) for the treatment of sTBI may result in intra- and post-operative complications. Controlled decompression may reduce these complications. Previous clinical trials in China have not yielded conclusive results regarding controlled decompression for sTBI. Therefore, we explored whether controlled decompression treatment decreases the rates of complications and improves the outcomes of patients with sTBI.

**Methods:** We performed this randomized, controlled trial at our hospital. Patients with sTBI aged 18–75 years old were randomly (1:1) divided into controlled decompression surgery (*n* = 124) or rapid decompression surgery groups (*n* = 124). The primary outcome measures were the Extended Glasgow Outcome Scale (GOS-E) score at 6 months and 30-days all-cause mortality. The secondary outcomes were the incidences of intraoperative brain swelling, post-traumatic cerebral infarction, and delayed hematoma.

**Results:** Compared with the rapid decompression group, the controlled decompression group had reduced 30-days all-cause mortality (18.6 vs. 30.8%, *P* = 0.035) and improved the 6-months GOS-E scores, and the difference was significant. In addition, the patients in the controlled decompression group had a lower intraoperative brain swelling rate (13.3 vs. 24.3%, *P* = 0.036), a lower delayed hematoma rate (17.7 vs. 29.0%, *P* = 0.048) and a relatively lower post-traumatic cerebral infarction rate (15.0 vs. 22.4%, *P* = 0.127) than those in the rapid decompression group.

**Conclusions:** Our data suggest that controlled decompression surgery significantly improves sTBI outcomes and decreases the rates of sTBI-related complications. However, this was a single-hospital study, and well-designed multicenter randomized controlled trials are needed to evaluate the effects of controlled decompression surgery for the management of patients with sTBI.

**Clinical Trial Registration:** Chinese Clinical Trial Registry; Date: 14/Dec/2013; Number: ChiCTR-TCC-13004002.

## Introduction

Traumatic brain injury (TBI) is among the most important public health problems; it has a significant influence on the lives of injured individuals and their family members and has high incidence and mortality rates (1, 2). Uncontrollable high intracranial pressure (ICP) may be the key to the poor outcomes in severe TBI (sTBI) patients (1, 3). Decompressive craniectomy (DC) is a means of rapidly reducing the ICP in patients with sTBI (1, 3, 4). Although the standard surgical method of DC (rapidly releasing the ICP) has been reported to effectively improve the prognosis in some studies (4, 5), a recent multinational, randomized trial has indicated that it may be associated with high rates of disability, mortality and post-operative complications (6). Cooper et al. (7) also reported that even though early bifrontotemporoparietal DC can decrease the ICP and the length of stay in the ICU for severe diffuse TBI and refractory ICP in adults, it was also associated with a significantly poorer outcome at 6 months according to the Extended Glasgow Outcome Scale (GOSE) score. Our previous studies found that rapid release of the ICP in sTBI patients can easily lead to acute intraoperative encephalocele, delayed hematoma, and post-operative cerebral infarction. In addition, a rapid decline in the ICP may result in subsequent ischemia-reperfusion injury and cerebral hemorrhage (8). The mechanism may be related to rapid reperfusion of the arterial circulation with continued obstruction of the venous outflow.

Controlled decompression in DC is an effective craniotomy method whereby the ICP is gradually released; all the steps are determined by the ICP throughout the surgical procedure (as opposed to rapid release of the ICP with conventional craniotomy). The cerebral arteries will lose cerebrovascular self-regulation, and the cerebral veins will be compressed, leading to reduced cerebral venous system blood return after the ICP increases enough to require craniotomy after sTBI (9–12). At that point, rapid craniotomy (opening the skull and dura quickly, without controlled ICP release) will cause large amounts of arterial blood to pour quickly into the brain tissue, but without appropriate venous outflow. Therefore, the role of controlled decompression is to gradually maintain the balance of brain blood inflow and outflow. Additionally, when the ICP is released rapidly, the brain stem is displaced, and the contralateral hematoma increases rapidly as the pressure decreases. Controlled decompression aims to minimize potential ischemia-reperfusion injury, acute intraoperative encephalocele, and post-operative cerebral infarction, thereby maximizing the protection of cerebral vascular and nerve function (8). Our hospital has focused on this method for over 10 years and has found that some specific techniques, such as controlled ventricular drainage and controlled hematoma evacuation, may improve the outcomes of sTBI patients. However, no high-quality randomized trials have compared the benefits of the two different surgical methods. As our previous clinical study was a relatively small sample preliminary study, the design of study was not so rigorous, and no strict exclusion criteria, which reduced statistical power. Therefore, we conducted a prospective, randomized, controlled trial to compare the efficacy of controlled decompression and rapid decompression after craniotomy for sTBI at our hospital.

## Methods

### Study Design

**Figure 1:**
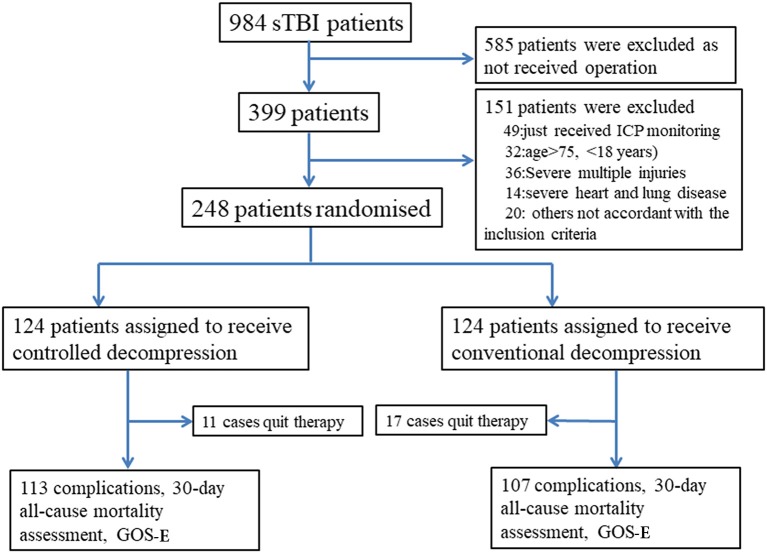
CONSORT diagram of the study.

We performed a randomized, controlled trial in our neurosurgical department at the Anhui Medical University-affiliated Wuxi Clinical College in Jiangsu, China, between January 1, 2014, and January 1, 2016. The study was designed to assess the superiority of the intervention. The study protocol was approved by the Anhui Medical University-affiliated Wuxi Clinical College Clinical Research Ethics Committee (2013-009). We obtained written informed consent from the family members of patients whose competence was established by their accurate orientation to time, place, and person and understanding of the recruiter's description of the trial. Otherwise, consent was obtained from the patient's next of kin or legal representative (Appendix). Patients were randomly assigned (1:1) to undergo controlled or rapid decompression after sTBI (Figure 1). We selected this randomization strategy to achieve equiveillance in the treatment groups. The final follow-up visit was 6 months after the sTBI.

### Study Patients

sTBI patients aged 18–75 years old were eligible if an indication for DC existed and a legally acceptable representative was able to provide informed consent. sTBI was defined as a Glasgow Coma Scale (GCS) score between 3 and 8 on admission. Indications for DC included preoperative diffuse brain swelling, large-volume preoperative hematoma (≥30 mL) and obvious compression of the brain tissue (deviation from the midline >1 cm, pressure, and distortion of the lateral ventricles and basal cistern). The exclusion criteria were as follows: brain swelling caused by anoxia or hypotension with minor intracranial bleeding after injury; coagulation disorder or a history of aspirin intake and multiorgan malfunction; special injury location, such as hematoma of the brain stem or ventricle; initial need for bilateral craniectomy; preoperative GCS score of 3 with no improvement after treatment in the emergency room; presentation without attenuated respiration and blood pressure; combination with severe injury in another bodily region; lack of consent from family members for participation in the clinical trial; and patient participation in other clinical trials.

### Randomization

Permuted-block randomization was performed using a computer system with an allocation list consisting of random numbers (at a 1:1 ratio) generated using SPSS 14.0 software (SPSS Institute, Hefei, Anhui Medical University) by a statistician not associated with the project team. During the study period, all included patients were randomly assigned to undergo either controlled or rapid decompression after sTBI before the operation.

### Craniectomy Method and Random Assignment

All patients underwent baseline cranial computed tomography (CT) and CT angiography (CTA) to assess changes in cerebral blood vessels and blood flow before the operation and to guide the surgery. According to the Chinese guidelines for TBI and our previous research (3), emergency craniotomy was indicated if the ICP (Codman, USA) continued to increase and was >25 mmHg after treatment with mannitol dehydration, sedation, and analgesia, if the GCS score decreased by >2 and CT re-examination showed that the contusions and hematomas had enlarged, and if the cisterna ambiens had disappeared, there was a midline shift, the ventricles were compressed, or similar features were present. If patients had a large hematoma and cerebral hernia, then ICP monitor placement and craniotomy were performed at the same time, with the ICP monitor being placed just prior to opening of the bone flap and dura. Two neurosurgeons together determined whether DC was needed. When both neurosurgeons confirmed the operation and the patients' family members provided consent, the patients were randomly assigned to undergo controlled or rapid decompression surgery.

### Procedures

#### Rapid Craniectomy

Rapid craniectomy was performed using standard operative procedures (8, 13). A standard large craniotomy (12 × 15 cm) completely opened the dura, and the ICP was released rapidly, completely and without control. All patients in this group received ICP monitoring. The rate of decrease in the ICP was not controlled throughout the operation, and the intraoperative surgical method did not consider the ICP. The goal of the operation was to remove the hematoma and brain contusion tissue as quickly as possible.

#### Controlled Decompression (Figure 2)

Controlled decompression was achieved as previously described (8). The aim of controlled decompression was to ensure gradual release of the ICP through the overall procedure by all types of methods. The rate of ICP decrease was 10–15 mmHg per 10 min (8). Briefly, an ICP probe was inserted to obtain the initial ICP before craniectomy. Ventricular intracranial pressure monitor was the best choice, the next was brain tissue monitor. If the initial ICP was >40 mmHg, then the cerebrospinal fluid (CSF) was gradually released until the ICP was 40 mmHg. Second, craniotomy with a bone window (12 × 15 cm) was required to pressurize the brain to avoid a rapid decrease in the ICP after the bone was removed. Third, the dura was opened with an incision that was generally no larger than 5 mm, which is often the diameter of the aspirator head. The hematoma and brain contusion tissue were slowly aspirated, gradually reducing the ICP. When the ICP was below 10 mmHg and there were no signs of bulging brain tissue, the dura was completely opened, and the hematoma or brain contusion tissue was then removed.

**Figure 2:**
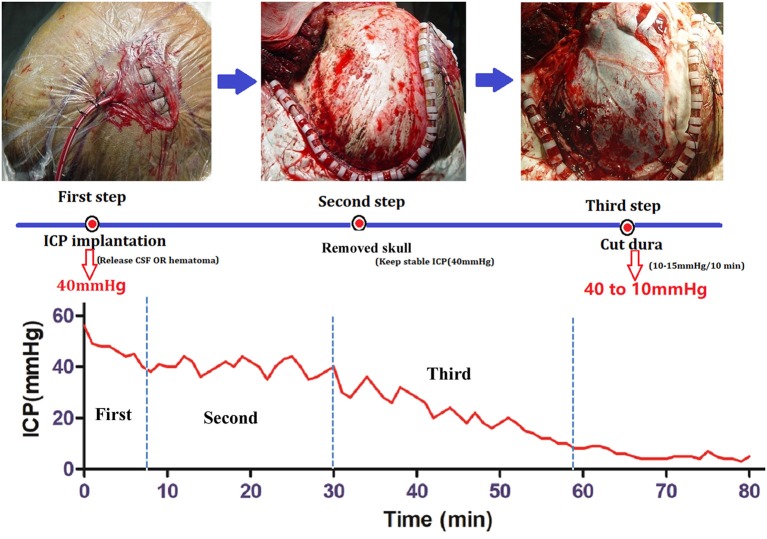
Procedure for controlled decompression and ICP monitoring. First step: ICP probe implantation. If the ICP was higher than 40 mmHg, then CSF was released under ventricular ICP monitoring until the ICP was 40 mmHg. Second step: Skull removal. Craniotomy was performed with a bone window 12 × 15 cm in size, and the ICP was maintained at 40 mmHg. Third Step: Dural incision and hematoma removal. The dura was opened with an incision that was generally no larger than 5 mm, and the hematoma and brain contusion tissue were slowly aspirated, gradually reducing the ICP.

#### Post-operative Treatment and Monitoring

After the operation, all patients were sent directly to the neurosurgical intensive care unit (NICU). The post-operative management strategy was selected by one neurosurgeon and one NICU doctor. The post-operative treatments were the same for the patients in both groups. Therapeutic hypothermia was performed for 7–10 days for patients who had a high ICP. An ICP sensor was routinely used to monitor the ICP post-operatively (the sensor was typically removed ~1 week after surgery). The vital signs and ICP were observed and recorded every 2 h. The cranial CT results were reviewed routinely at 1, 24, and 72 h after the operation if the patients were stable.

#### Outcome Assessment

The primary endpoints were the 30-days all-cause mortality rate and the post-operative neurological outcome, as determined by the Extended Glasgow Outcome Scale (GOSE) score (14, 15) assessed at 6 months after injury via a face-to-face interview with two neurosurgeons (blind investigators in this study). The GOSE score is a global measure of function and health-related quality of life and was graded according to the following 3 levels: favorable (GOSE score 5–8), unfavorable (GOSE score 2–4), and dead (GOSE score 1).

Operative complications were assessed by comparing the occurrence of delayed hematoma, acute operative brain swelling, and cerebral infarction between the two groups. Delayed hematoma was indicated when intracranial hematoma was not detected on the initial or post-operative CT scan but was detected on a subsequent scan. Acute operative brain swelling was indicated by brain tissue bulging from the bone defect area because of an acute increase in the ICP during surgery or by a post-operative CT scan showing brain swelling combined with an increased ICP.

Cerebral infarction, diagnosed by CT at the time of hospitalization, was defined as an ischemic brain lesion that formed after the operation. On CT, cerebral infarction was indicated by abnormal hypodense areas with clear margins in regions with an arterial blood supply. In contrast, cerebral contusion was indicated by areas of heterogeneous density in the affected region. Where necessary, CTA or perfusion CT was performed to confirm the diagnosis of cerebral infarction. Transcranial Doppler (TCD) was also used daily to evaluate cerebral blood flow.

### Statistical Analysis

A research nurse entered all baseline and outcome data in the study database. Data were collected on handwritten forms and archived in a password-protected, electronic database. All continuous variables are presented as the mean ± standard deviation. SPSS 14.0 statistical software (SPSS, Inc., Chicago, USA) was used for the statistical analyses. Independent two-sample *t*-tests and Spearman correlations were used to assess categorical data. Fisher's exact *t*-test was used to compare categorical data between two groups, and the Mann-Whitney *U* test was used to compare ordinal or continuous variables between groups. A value of *P* < 0.05 was considered statistically significant. In our pilot study, 15% of the patients in the controlled decompression surgery group died, compared with 30% in the rapid decompression group. On the basis of these data, we estimated that 118 patients would be needed to confirm this effect with an α of 5 and 80% power. To allow the reliable detection of a slightly smaller effect and account for a 5% loss to follow-up rate, we decided to enroll 248 patients.

## Results

Between January 1, 2014, and January 1, 2016, 984 sTBI patients were assessed, and 248 were randomly assigned to undergo either controlled (*n* = 124) or rapid decompression (*n* = 124) surgery (Figure 1). The baseline characteristics of the patients were not significantly different between the controlled and rapid decompression groups (Table 1). All patients were included in the final intention-to-treat analyses, except for 11 patients who quit therapy in the controlled decompression group and 17 patients who quit therapy in the rapid decompression group (Figure 1). All of these 28 patients quit therapy because the family members declined treatment, and the patients were discharged from the hospital, with no difference between the two groups. The final visit of the last randomized patient occurred on August 20, 2017.

**Table 1:** Demographic and baseline characteristics of the study population in two group.

**Variable**	**Controlled decompression**	**Conventional decompression**	***P*-value**
Number of patients	124	124	
Age (mean ± SD)	48.0 ± 13.5	50.3 ± 14.6	0.191
Gender			0.473
Male	94 (75.8%)	88 (71.0%)	
Female	30 (24.2%)	36 (29.0%)	
GCS at admission			0.429
3–5	49 (39.5%)	42 (33.9%)	
6–8	75 (60.5%)	82 (66.1%)	
Intubation	26 (21.0%)	21 (16.9%)	0.517
Hypovolemic shock	14 (11.3%)	17 (13.7%)	0.702
Time (from TBI to DC)	12.0 ± 5.9	12.4 ± 7.1	0.479
Rotterdam CT score at admission			0.268
I–II	21 (16.9%)	26 (21.0%)	
III–IV	46 (37.1%)	49 (39.5%)	
V–VI	57 (46.0%)	49 (39.5%)	
Mechanism of injury			0.534
Traffic of accident	81 (65.3%)	86 (69.4%)	
Fall	18 (14.5%)	15 (12.1%)	
Others	25 (20.2%)	23 (18.5%)	
Mydriasis			0.302
No	23 (18.5%)	27 (21.8%)	
Single	46 (37.1%)	50 (40.3%)	
Bilateral	55 (44.4%)	47 (37.9%)	
Type of hematoma*			0.974
Epidural	44 (35.5%)	39 (31.5%)	
Subdural	57 (46.0%)	60 (48.4%)	
Intracerebral	68 (54.8%)	62 (50.0%)	
Initial ICP (mean ± SD)	41.4 ± 13.5	40.9 ± 12.9	0.803
Hypothermia	71 (57.3%)	78 (62.9%)	0.488

GCS, Glasgow Coma Scale. ICP, Intracranial Pressure.

Type of hematoma*: 45 patients in the controlled decompression group and 37 patients in the decompressive craniectomy group had multiple types of hematoma.

### Demographic and Clinical Data

The patient demographics before surgery, the cause of the injury, the mydriasis status, age, sex, and the initial GCS score are summarized for the two groups (Table 1). There were no significant differences between the two groups in terms of the baseline data or initial ICP (Table 1, 41.4 ± 13.5 vs. 40.9 ± 12.9, *P* = 0.803).

### Primary Endpoint: Clinical Outcomes

According to the 30-days all-cause mortality results, 21 (18.6%) of 113 patients in the controlled decompression group and 32 (30.0%) of the 107 patients in the rapid decompression group died within 30 days (OR 0.512, *P* = 0.035, 95% CI 0.273–0.958). The 30-days all-cause mortality distribution was significantly different between the controlled and rapid decompression groups. Based on these data, undergoing controlled decompression surgery is associated with an ~11% lower risk of 30-days all-cause mortality (Table 2). At 6 months, there were significant differences in the GOSE classifications between the two groups (Table 2). The controlled decompression group showed higher GOSE scores and better outcomes than the rapid decompression group (Table 2).

**Table 2:** Comparison of endpoint-clinical outcomes between the two groups.

**Variable**	**Controlled decompression**	**Conventional decompression**	***P*-value**
Number of patients	113	107	
GOS-E			0.032
Favorable (5–8 score)	48 (42.5%)	33 (30.8%)	
Unfavorable (2–4 score)	34 (30.1%)	31 (29.0%)	
Dead (1 score)	31 (27.4%)	43 (40.2%)	
30-days All-cause mortality	21 (18.6%)	32 (30.8%)	0.035

*Data are presented as numbers (%) and were compared between groups using the Pearson Chi-square test and rank sum test*.

### Secondary Endpoint: Post-operative Complications

The occurrence of intraoperative brain swelling/acute encephalocele was significantly lower in the controlled decompression group than in the rapid decompression group (Table 3, 13.3 vs. 24.3%, *P* = 0.036). In the controlled and rapid decompression groups, 66.7% of 15 and 73.1% of 26 intraoperative brain swelling patients died, respectively.

**Table 3:** Comparison of post-operative complications between the two groups.

**Variable**	**Controlled decompression**	**Conventional decompression**	***P*-value**
Number of patients	113	107	
Delayed hematoma	20 (17.7%)	31 (29.0%)	0.048^*^
Acute brain swelling	15 (13.3%)	26 (24.3%)	0.036^*^
Cerebral infarction	17 (15.0%)	24 (22.4%)	0.127

Data are presented as numbers (%) and were compared between groups using the Pearson Chi-square test.

* *Indicates a statistically significant between groups difference (P < 0.05)*.

Delayed hematoma is a very important post-operative complication of contrecoups or injury-related cerebral contusions and can contribute significantly to acute encephalocele. In this study, 17.7% (20/113) of patients in the controlled decompression group and 29% (31/107) of patients in the rapid decompression group developed delayed hematoma (Table 3, *P* = 0.048).

Post-traumatic cerebral infarction (PTCI) is one of the most severe secondary insults after TBI. There was no significant difference in the incidence of PTCI between the groups. The reason may be the small sample size. However, the risk of PTCI was 7.5% less in the controlled decompression group than in the rapid decompression group (Table 3, *P* = 0.127).

## Discussion

Our randomized, single-blind, controlled trial results show that controlled decompression surgery significantly decreases the incidence of delayed hematoma and acute brain swelling and leads to improved outcomes. There was a significant tendency toward reduced mortality and improved outcomes with controlled DC. We found that patients in the controlled decompression group had better outcomes at 6 months after injury than those in the rapid decompression group. Furthermore, fewer patients in the controlled decompression group than in the rapid decompression group died within 30 days. Bao et al. (6) reported that good recovery was observed in 21.6% of patients with sTBI and malignant diffuse brain swelling, while 18.9% of the patients died. A recent international, multicenter, parallel-group, randomized, superiority trial (16) showed that 26.9% of 201 sTBI patients with refractory elevated ICP (>25 mmHg) in the surgical group and 48.9% of 188 patients in the medical group died, with good recovery observed in only ~10% of patients. Therefore, in our rapid decompression group, the proportion of patients who showed good recovery and the mortality rate were within the ranges reported in the literature for patients with sTBI who were treated with DC. However, in this randomized trial, better results were observed with controlled decompression. The major reasons might be related to post-operative complications.

In this study, we found that the incidence of delayed hematoma was 29% in the rapid decompression group and only 17.7% in the controlled decompression group. Therefore, controlled decompression and surgical technology can significantly reduce the incidence of delayed hematoma. We previously reported that more than 20% of sTBI patients developed delayed hematoma after surgery, and most of them may require a second operation to evacuate the hematoma (8). Most researchers consider post-operative hemorrhage a well-known and rare but serious complication of intracranial procedures that usually occurs at the operation site but may also occur remotely; contralateral epidural/subdural hematoma is also possible (17, 18). Huang et al. (17) reported that the incidence of remote epidural hemorrhage (EDH) following decompressive hemicraniectomy for TBI was 7.9%. As previous studies of these complications are mostly case reports, they lack complete cohort and follow-up analyses and include all TBI patients, not just sTBI patients; thus, the reported incidence of delayed contralateral hematoma is lower in these studies than in our study. The exact mechanism of contralateral hematoma occurring after decompression surgery remains uncertain. The most widely accepted hypothesis is that decompression surgery leads to the acute release of tamponade on the contralateral bleeding source, causing the development of contralateral hematoma; another important factor is that contralateral calvarial fractures can lead to contralateral epidural hematoma following DC (19–21). Our experiences indicate that there is a greater risk of delayed contralateral hematoma if the ICP is reduced rapidly, like rapid decompression. In contrast, the incidence of delayed contralateral hematoma decreased significantly if the ICP was released slowly. Delayed contralateral epidural hematoma may present as intraoperative brain swelling, post-operative pupillary abnormalities, intractably increased ICP, or neurological worsening. If not diagnosed in a timely manner, contralateral epidural hematoma can have serious consequences (17, 21, 22).

Intraoperative brain swelling is a very common and intractable problem in sTBI patients treated with craniectomy. Our previous study examined 545 sTBI patients and revealed acute brain swelling in 24.2% of patients; most of these patients with intraoperative brain swelling died (23). In this study, 15 patients in the controlled decompression group and 26 in the rapid decompression group exhibited acute brain swelling. Therefore, the incidence in the controlled decompression group was 11% less than that in the rapid decompression group. The exact mechanism of brain swelling in craniectomy also remains uncertain, but the most important and common reason is delayed contralateral hematoma (8, 17, 19–23). Additionally, Langfitt et al. (24) indicated that the mechanism may be related to cerebrovascular dilatation and increased cerebral blood volume after craniectomy or the rapid release of ICP. Therefore, controlled decompression was more effective for preventing the development of delayed hematoma, reducing shifts of the brainstem, and decreasing the incidence of acute intraoperative encephalitis (8, 23, 25).

PTCI is one of the most severe secondary insults after TBI, and it has been presented as an indicator of poor clinical outcomes, with a high mortality rate despite appropriate medical and surgical interventions (8, 26–28). Some studies have reported that the overall mortality rate is as high as 50% (28, 29). We found that the incidence of post-operative cerebral infarction was higher in the rapid decompression group than in the controlled decompression group. Although many authors have reported that PTCI is rarely observed in TBI (27, 29), it is very common in sTBI patients who undergo DC (8, 25, 28). Sauvigny et al. (28) reported that 57 patients undergoing DC because of a space-occupying middle cerebral artery infarction developed PTCI. Su et al. (25) also reported that the incidence of PTCI following DC was 31.2%. There are many mechanisms that may be involved in PTCI, including cerebrovascular injury, ischemia-reperfusion injury, direct vascular compression due to intracranial hematoma, brain edema, cerebral vasospasm, thromboembolism, and systemic hypoperfusion (8, 14). We suggest that controlled decompression is a good method for achieving the gradual recovery of cerebral blood flow/volume and alleviating ischemia-reperfusion injury. The incidence of PTCI in the controlled decompression group was 7.4% less than that in the rapid decompression group. Additional multicenter studies involving larger patient groups are needed to confirm and extend our findings.

Our study is limited by the relatively small number of patients in each group, which reduced the statistical power of the analysis. Additionally, this study was performed at a single center. We suggest that a similar, better-controlled, larger-scale, multicenter study is needed to confirm the encouraging results of our study (Chinese Clinical Trial Registry, ChiCTR1800016909).

## Conclusion

In summary, we found that the 6-months outcomes and the incidence rates of intraoperative acute brain swelling and delayed intracranial hematoma were significantly better in sTBI patients who underwent controlled decompression than in those who underwent rapid decompression. Although we were unable to demonstrate statistical significance for the difference in the PTCI rate, a greater proportion of patients who underwent controlled decompression showed good post-operative outcomes. Furthermore, the incidence of PTCI and the 30-days all-cause mortality rate were lower with controlled decompression than with rapid decompression. Our preliminary findings suggest that controlled decompression may be a very good method and concept for preventing intraoperative acute brain swelling, delayed intracranial hematoma and PTCI. Nevertheless, a larger, multicenter, controlled study is needed to clarify whether this method improves patient prognosis.

## Data Availability Statement

The datasets generated for this study are available on request to the corresponding author.

## Ethics Statement

The study protocol was approved by the Anhui Medical University-Affiliated Wuxi Clinical College Clinical Research Ethics Committee (2013-009). We obtained written informed consent from the family members of patients whose competence was established by accurate orientation to time, place, and person and understanding of the recruiter's description of the trial. Otherwise, consent was obtained from the patient's next of kin or legal representative (Appendix).

## Author Contributions

JC, YW, and QC designed this research. LC, CZ, WC, and YF collected the relevant literatures. JC and ML wrote and revised the manuscript. All authors have read and approved the final manuscript.

### Conflict of Interest

The authors declare that the research was conducted in the absence of any commercial or financial relationships that could be construed as a potential conflict of interest.

## Abbreviations

CT: computed tomography
CTA: computed tomography angiography
CTP: computed tomography perfusion
CSF: cerebrospinal fluid
DC: decompressive craniectomy
GCS: Glasgow Coma Scale
GOSE: Extended Glasgow Outcome Scale
ICP: intracranial pressure
NICU: neurosurgical intensive care unit
PTCI: post-traumatic cerebral infarction
sTBI: severe traumatic brain injury
TCD: transcranial Doppler.
