# Supplementary material for: The Effect of Controlled Decompression for Severe Traumatic Brain Injury: A Randomized, Controlled Trial
Source: Front Neurol. 2020 Feb 18;11:107. doi: 10.3389/fneur.2020.00107 (PMC7040242; doi:10.3389/fneur.2020.00107)
Supplement: Supplementary file 1 [file Table_1.DOC]

Patient Consent Letters

Dear patients and your family members:

The doctor had confirmed that you are: **severe traumatic brain injury and need operation**. We will invite you to participate in a clinical study on the treatment of severe traumatic brain injury, Public title: **Randomized, Single-blind, Controlled Study to Evaluate the Efficacy of Controlled Decompression during Severe Traumatic Brain Injury Craniotomy**. Registration number: **ChiCTR-TCC-13004002**. This research proposal has been reviewed by the medical ethics committee of the 101^th^ hospital of the people's liberation army and agreed to conduct clinical research.

Before you decide whether to participate in this study, please read the following as carefully as possible. It can help you understand the study and why it was conducted, the procedures and duration of the study, and the possible benefits, risks, and discomforts of participating in the study.

If you wish, you can discuss it with your relatives and friends, or ask your doctor for an explanation to help you make a decision.

I. Research introduction and purpose

1. Introduction

Traumatic brain injury (TBI) is among the most important public health problems; it has a significant influence on the lives of injured individuals and their family members and has high incidence and mortality rates. Uncontrollable high intracranial pressure (ICP) may be the key to the poor outcomes in sTBI patients. Decompressive craniectomy (DC) is a means of rapidly reducing the ICP in patients with sTBI. Although the standard surgical method of DC (rapidly releasing the ICP) has been reported to effectively improve the prognosis in some studies, a recent multinational, randomized trial has indicated that it may be associated with high rates of disability, mortality and postoperative complications. Cooper also reported that even though early bifrontotemporoparietal DC can decrease the ICP and the length of stay in the ICU for severe diffuse TBI and refractory ICP in adults, it was also associated with a significantly poorer outcome at 6 months according to the Extended Glasgow Outcome Scale (GOSE) score. Our previous studies found that rapid release of the ICP in sTBI patients can easily lead to acute intraoperative encephalocele, delayed hematoma, and postoperative cerebral infarction. In addition, a rapid decline in the ICP may result in subsequent ischemia-reperfusion injury and cerebral hemorrhage. The mechanism may be related to rapid reperfusion of the arterial circulation with continued obstruction of the venous outflow.

Controlled decompression in DC is an effective craniotomy method whereby the ICP is gradually released; all the steps are determined by the ICP throughout the surgical procedure (as opposed to the rapid release of the ICP with conventional craniotomy). The cerebral arteries will lose cerebrovascular self-regulation, and the cerebral veins will be compressed, leading to reduced cerebral venous system blood return after the ICP increases enough to require craniotomy after sTBI. At that point, rapid craniotomy (opening the skull and dura quickly, without controlled ICP release) will cause large amounts of arterial blood to pour quickly into the brain tissue, but without appropriate venous outflow. Therefore, the role of controlled decompression is to gradually maintain the balance of brain blood inflow and outflow. Additionally, when the ICP is released rapidly, the brain stem is displaced, and the contralateral hematoma increases rapidly as the pressure decreases. Controlled decompression aims to minimize potential ischemia-reperfusion injury, acute intraoperative encephalocele, and postoperative cerebral infarction, thereby maximizing the protection of cerebral vascular and nerve function. Our hospital has focused on this method for over 10 years and has found that some specific techniques, such as controlled ventricular drainage and controlled hematoma evacuation, may improve the outcomes of sTBI patients. However, no high-quality randomized trials have compared the benefits of the two different surgical methods.

2. Research purpose

we conducted a prospective, randomized, controlled trial to compare the efficacy of controlled decompression and rapid decompression after craniotomy for sTBI at our hospital.

1. Participating hospital and an expected sample of participants

Chinese 101^th^ hospital of the people's liberation army, n=250

II. The exclusion criteria:

brain swelling caused by anoxia or hypotension with minor intracranial bleeding after injury; coagulation disorder or a history of aspirin intake and multiorgan malfunction; special injury location, such as hematoma of the brain stem or ventricle; initial need for bilateral craniectomy; preoperative GCS score of 3 with no improvement after treatment in the emergency room; presentation without attenuated respiration and blood pressure; combination with severe injury in another bodily region; lack of consent from family members for participation in the clinical trial; and patient participation in other clinical trials.

III. What will you need to do if you participate in the study?

1. Before you are enrolled in the study, your doctor will have some questions and record your medical history, and you must have a **CT/CTA examinations**. You are eligible to participate in the study, then sign the informed consent. If you do not wish to participate in the study, we will treat you as you wish.
2. If you are willing to participate in the study, the following steps will be followed:

Briefly, an ICP probe was inserted to obtain the initial ICP before craniectomy. If the initial ICP was >40 mmHg, then the cerebrospinal fluid (CSF) was gradually released until the ICP was 40 mmHg. Second, craniotomy with a bone window (12 cm*15 cm) was required to pressurize the brain to avoid a rapid decrease in the ICP after the bone was removed. Third, the dura was opened with an incision that was generally no larger than 5 mm, which is often the diameter of the aspirator head. The hematoma and brain contusion tissue were slowly aspirated, gradually reducing the ICP. When the ICP was below 10 mmHg and there were no signs of bulging brain tissue, the dura was completely opened, and the hematoma or brain contusion tissue was then removed. Then follow-up was carried out routinely to check whether there were operation-related complications.

IV. Possible benefits of a participator in research

Although there is some evidence confirmed that controlled decompression surgery had a satisfactory outcome for severe TBI, but there is no guarantee that it will good for you. The prospective study used in this study is not the only treatment for severe TBI, If controlled decompression surgery does not good for your condition, ask your doctor to choose a possible alternative treatments.

V. Adverse reactions, risks, and inconveniences

It may also cause the condition aggravation, no effect, and increased risk of intracranial infection or hydrocephalus.

VI. Expenses

If patients occurred adverse reactions, the investigator and hospital will pay the cost of expenses to treat the adverse reactions and the patient's potential compensation.

The doctor will do his best to prevent and treat any possible harm as a result of this study. If an adverse event occurs in a clinical trial, a committee of medical experts will determine whether it is related to the operation. The sponsor will provide the cost of treatment and corresponding economic compensation for the injury related to the test in accordance with the provisions of the Chinese clinical trial quality management standard. The treatment and examination required for other diseases that you are co-existing with will not be free of charge.

VII. Confidentiality of personal information

Your medical records (study records /CRF, lab tests, etc.) will be kept intact at the hospital you are attending.Your doctor will record the results of tests and other [image logical](../../../../C:%5CUsers%5CAdministrator%5CAppData%5CLocal%5Cyoudao%5CDictBeta%5CApplication%5C7.5.2.0%5Cresultui%5Cdict%5C%3Fkeyword=imageological)[examination](../../../../C:%5CUsers%5CAdministrator%5CAppData%5CLocal%5Cyoudao%5CDictBeta%5CApplication%5C7.5.2.0%5Cresultui%5Cdict%5C%3Fkeyword=examination) on your medical record. Researchers, ethics committees, and drug regulators will be allowed access to your medical records. Any public report on the results of this study will not disclose your personal identity. We will make every effort to protect the privacy of your personal medical data to the extent permitted by law.

According to medical research ethics, apart from personal privacy information, the trial data will be available for public inquiry and sharing, which will be limited to web-based electronic databases to ensure that no personal privacy information will be disclosed.

VIII. How to get more information?

You can ask any questions about this study at any time and get answers accordingly. Your doctor will keep you informed of any important new information that may affect your willingness to continue the study.

IX. You can choose to participate in the study and withdraw from the study

Whether to participate in the study is entirely up to you. You may refuse to participate in the study or withdraw from the study at any time during the study, which will not affect your relationship with your doctor or affect the loss of your medical or other benefits. In the best interest of you, the physician or investigator may discontinue your participation at any time during the study. If you quit from the study for any reason, you may also be asked for laboratory and physical tests if your doctor thinks so.

X. What to do now?

Whether to participate in this study is up to you (or your family).

Ask your doctor as many questions as possible before you make the decision to participate in the study.

Thank you for reading the above materials. If you decide to participate in this study, please tell your doctor, he/she will arrange everything related to the study for you.

Please keep this information.

**Agree with the statement**

I have read the above introduction to this study and had the opportunity to discuss and ask questions about this study with my doctor. All my questions have been answered satisfactorily.

I am aware of the possible risks and benefits of participating in this study.

I understand that participation in the study is voluntary and I confirm that I had sufficient time to consider it.

Participant signature Date

Tel:

I confirm that I have explained the details of the trial to the patient, including its rights and possible benefits and risks, and have given them a signed copy of the informed consent.

Doctor signature Date

Tel:
